# Supplementary material for: Vitamin D levels and its influencing factors in pregnant women in mainland China: A systematic review and meta-analysis
Source: PLoS One. 2024 May 9;19(5):e0297613. doi: 10.1371/journal.pone.0297613 (PMC11081274; doi:10.1371/journal.pone.0297613)
Supplement: Attachment — Submitted filename: Response to Reviewers_.docx [file pone.0297613.s003.docx]

Dear Editor and Reviewers:

Thanks for your letter and for the reviewers’ comments concerning our manuscript entitled “Vitamin D Levels and Its Influencing Factors in Pregnant Women in Mainland China：a systematic review and meta-analysis” (ID: PONE-D-23-31837). Those comments are all valuable and very helpful for revising and improving our paper, as well as the important guiding significance to our researches. We have studied comments carefully and have made correction which we hope meet with approval. Revised portion are marked in red in the paper. The main corrections in the paper and the responds to the reviewer’s comments are as following:

**The academic editor:**

Journal Requirements:

When submitting your revision, we need you to address these additional requirements.

1. Please ensure that your manuscript meets PLOS ONE's style requirements, including those for file naming. The PLOS ONE style templates can be found at
   [https://journals.plos.org/plosone/s/file?id=wjVg/PLOSOne_formatting_sample_main_body.pdf](https://journals.plos.org/plosone/s/file?id=wjVg/PLOSOne_formatting_sample_main_body.pdf) and
   [https://journals.plos.org/plosone/s/file?id=ba62/PLOSOne_formatting_sample_title_authors_affiliations.pdf](https://journals.plos.org/plosone/s/file?id=ba62/PLOSOne_formatting_sample_title_authors_affiliations.pdf)

**Response:** Thank you very much for your reminder. We have checked it again, and ensured that the manuscript meet PLOS ONE’s style requirements, including those for file naming.

2. PLOS requires an ORCID iD for the corresponding author in Editorial Manager on papers submitted after December 6th, 2016. Please ensure that you have an ORCID iD and that it is validated in Editorial Manager. To do this, go to ‘Update my Information’ (in the upper left-hand corner of the main menu), and click on the Fetch/Validate link next to the ORCID field. This will take you to the ORCID site and allow you to create a new iD or authenticate a pre-existing iD in Editorial Manager. Please see the following video for instructions on linking an ORCID iD to your Editorial Manager account: [https://www.youtube.com/watch?v=_xcclfuvtxQ](https://www.youtube.com/watch?v=_xcclfuvtxQ)

**Response:** Thank you very much for your reminder. The ORCID iD for the corresponding author (Zisheng Li,) is0009-0005-7807-0046 , and it is validated in Editorial Manager.

3. Please include captions for your Supporting Information files at the end of your manuscript, and update any in-text citations to match accordingly. Please see our Supporting Information guidelines for more information: [http://journals.plos.org/plosone/s/supporting-information.](http://journals.plos.org/plosone/s/supporting-information.)

**Response:** Thank you very much for your reminder. The captions for the Supporting Information file was added in the revised manuscript.

“**Supporting Information:** PRISMA 2020 checklist.”

[Note: HTML markup is below. Please do not edit.]

**Reviewers' comments:**

Reviewer's Responses to Questions

**Comments to the Author**

1. Is the manuscript technically sound, and do the data support the conclusions?

The manuscript must describe a technically sound piece of scientific research with data that supports the conclusions. Experiments must have been conducted rigorously, with appropriate controls, replication, and sample sizes. The conclusions must be drawn appropriately based on the data presented.

Reviewer #1: Yes

Reviewer #2: Yes

Reviewer #3: Yes

2. Has the statistical analysis been performed appropriately and rigorously?

Reviewer #1: N/A

Reviewer #2: Yes

Reviewer #3: Yes

3. Have the authors made all data underlying the findings in their manuscript fully available?

The [PLOS Data policy](http://www.plosone.org/static/policies.action#sharing) requires authors to make all data underlying the findings described in their manuscript fully available without restriction, with rare exception (please refer to the Data Availability Statement in the manuscript PDF file). The data should be provided as part of the manuscript or its supporting information, or deposited to a public repository. For example, in addition to summary statistics, the data points behind means, medians and variance measures should be available. If there are restrictions on publicly sharing data—e.g. participant privacy or use of data from a third party—those must be specified.

Reviewer #1: Yes

Reviewer #2: Yes

Reviewer #3: Yes

4. Is the manuscript presented in an intelligible fashion and written in standard English?

PLOS ONE does not copyedit accepted manuscripts, so the language in submitted articles must be clear, correct, and unambiguous. Any typographical or grammatical errors should be corrected at revision, so please note any specific errors here.

Reviewer #1: Yes

Reviewer #2: Yes

Reviewer #3: Yes

5. Review Comments to the Author

Please use the space provided to explain your answers to the questions above. You may also include additional comments for the author, including concerns about dual publication, research ethics, or publication ethics. (Please upload your review as an attachment if it exceeds 20,000 characters)

**Reviewer #1:** In this study, the authors performed a systematic review and meta-analysis to comprehensively understand the prevalence of Vitamin D deficiency among pregnant women in mainland China. This study will provide some guidance for vitamin D supplementation of pregnant women. I have several questions as following:
1. The author also mentioned that the prevalence of vitamin D deficiency or insufficiency decreased during pregnancy progression. The author showed that the distinction in vitamin D deficiency or insufficiency during pregnancy among different regions and seasons is significant, as shown in Fig. 3 and Fig. 5, respectively. Could the authors clarify which pregnancy period’s vitamin D deficiency or insufficiency is significantly different among different regions and seasons?

**Response:** Thank you for your comment and question regarding the distinction in vitamin D deficiency or insufficiency during pregnancy among different regions and seasons. After carefully re-evaluating our findings and figures, we would like to clarify that our study did not specifically examine the significance of vitamin D deficiency or insufficiency during different periods of pregnancy among different regions and seasons. Our analysis focused on investigating the overall prevalence of vitamin D deficiency or insufficiency in pregnant women in mainland China. We acknowledge that there may be variations in vitamin D levels during different stages of pregnancy and among different regions and seasons. However, due to limitations in the available data and the scope of our study, we were unable to conduct a detailed analysis of these specific factors.We have addressed this issue in the limitations section of our paper. Thank you for bringing this to our attention, and we appreciate your understanding.

**In the eighth part of the discussion:** “Finally,due to limitations in the available data and the scope of our study, our study did not specifically examine the significance of vitamin D deficiency or insufficiency during different periods of pregnancy among different regions and seasons*.*”

2. How did the author define vitamin D deficiency or insufficiency? Since the requirement of vitamin D changes during pregnancy progression, did the author use different standards of vitamin D levels in different pregnancy periods?

**Response:** Thanks very much for reminder. The define vitamin D deficiency or insufficiency was added in the revised manuscript. In our study, we have defined the criteria for vitamin D deficiency or insufficiency and classified the pregnant women participating in the study accordingly. We based our definitions on the "Guidelines for the Application of Vitamin D in Adults' Bone Health" developed by the Vitamin D Discipline Group of the Osteoporosis Committee of the Chinese Geriatrics Society in 2014. According to these guidelines, a serum 25(OH)D level below 30 nmol/L is considered as vitamin D deficiency, and a level between 30-50 nmol/L is considered as vitamin D insufficiency,and vitamin D levels above 50 nmol/L are considered sufficient.

In our study, we used a standardized criterion to define vitamin D deficiency or insufficiency across all stages of pregnancy, without considering different criteria based on the progression of pregnancy. This approach was chosen because our main objective was to investigate the overall vitamin D status of pregnant women, rather than analyzing the variation in vitamin D requirements during specific stages of pregnancy.

However, we acknowledge that the vitamin D requirements may indeed vary during different stages of pregnancy. In future research, it would be valuable to consider the progression of pregnancy and relevant guidelines in order to assess vitamin D deficiency or insufficiency using different criteria for each stage of pregnancy.

**In the second part of the materials and methods:** “We based our definitions on the "Guidelines for the Application of Vitamin D in Adults' Bone Health" developed by the Vitamin D Discipline Group of the Osteoporosis Committee of the Chinese Geriatrics Society in 2014. According to these guidelines, a serum 25(OH)D level below 30 nmol/L is considered as vitamin D deficiency, and a level between 30-50 nmol/L is considered as vitamin D insufficiency,and vitamin D levels above 50 nmol/L are considered sufficient*.*”

3. The author indicated that the difference in vitamin D levels among different pregnant women at different gestational stages was significant (P<0.05). However, Fig. 6 indicates that P=0.05. Please double check this inconsistency.

**Response:** Thank you very much for your care, and we apologize for our mistake. We have checked and revised the sentence in the revised manuscript.

**In the sixth part of the result:** “The difference in vitamin D levels among different pregnant women at different gestational stages was statistically significant (χ2=6.18,P=0.05), with forest plot referring to Fig 6.”

4. As the authors mentioned in discussion, maternal vitamin D status can be influenced by several factors, such as ethnicity, education level, BMI, sun exposure, pregnancy season, and usage of vitamin D supplements. Do the author have data to support the potential contribution of these factors to the significant differences observed in this study? The author should discuss more details in this regard.

**Response:** Thanks for your reminder. We appreciate your suggestion to delve further into these aspects in our study. However, it's important to note that our current research did not collect specific data related to these factors, and therefore, we are unable to provide empirical evidence to support their contribution to the significant differences observed in our study.

We agree that understanding the interplay of these variables could provide valuable insights into the variations in maternal vitamin D status. In future research, we plan to investigate these factors more comprehensively to elucidate their roles in influencing vitamin D levels during pregnancy. Your comment has highlighted an important avenue for further investigation, and we appreciate your valuable input.We have discussed this aspect in the revised manuscript.

**In the first part of the discussion:**“It's important to note that our current research did not collect specific data related to these factors, and therefore, we are unable to provide empirical evidence to support their contribution to the significant differences observed in our study.”

5. Genetic factor may also contribute to the differences observed in this study. The author should discuss it.

**Response:** Thanks for your good suggestion. We have added a paragraph for clarification in the second part of discussion.

**In the second part of the discussion:** “Genetic factors also influence maternal vitamin D during pregnancy. Sampathkumar et al. reported a genome-wide association analysis (GWAS) of vitamin D in maternal and fetal blood circulation. The GWAS analysis revealed that a missense variant, rs4588, and its associated haplotype in the population-specific component gene encoding Vitamin D binding protein (VDBP), serve as risk factors for prenatal vitamin D deficiency and low umbilical cord blood vitamin D levels. Furthermore, a novel association was discovered between a downstream SNP of the CYP2J2 gene (rs10789082), involved in vitamin D 25-hydroxylation, and maternal vitamin D, but this association was not observed in their offspring.”

Special thanks to you for your good comments.

**Reviewer #2:** This article is titled “Vitamin D Levels and Its Influencing Factors in Pregnant Women in Mainland China：a systematic review and meta-analysis” analyzed the VD deficiency during pregnancy in multiple angles, which is very interesting and meaningful, filling the knowledge gap in this field regarding the Chinese population in Mainland China. However, I still have some concerns that I hope the authors can address for publication:

1. The chemical properties of vitamin D should be introduced.

**Response**: Thanks very much for your good suggestion. We have added some sentences to introduce the chemical properties of vitamin D in the first part of the introduction.

**In the first part of the introduction:** “The active form of vitamin D in the body is known as 1,25-dihydroxyvitamin D. In the human body, vitamin D3 and D2 bind to vitamin D binding protein in the blood plasma and are transported to the liver, where they undergo 25-hydroxylation to become 25-hydroxyvitamin D [25(OH)D].”

2. What is the normal/healthy range of VD during pregnancy? What is the difference between “insufficiency” and “deficiency”?

**Response:** Thanks very much for reminder. Currently, there are no specific standards for the normal/healthy range of vitamin D in pregnant women; therefore, our criteria are based on those established for healthy adults.The define vitamin D normal/health range during pregnancy, deficiency or insufficiency was added in the revised manuscript.

**In the second part of the materials and methods:** “We based our definitions on the "Guidelines for the Application of Vitamin D in Adults' Bone Health" developed by the Vitamin D Discipline Group of the Osteoporosis Committee of the Chinese Geriatrics Society in 2014. According to these guidelines, a serum 25(OH)D level below 30 nmol/L is considered as vitamin D deficiency, and a level between 30-50 nmol/L is considered as vitamin D insufficiency,and vitamin D levels above 50 nmol/L are considered sufficient*.*”

3. Due to the VD deficiency during pregnancy, were there any consequences or symptoms shown on the offspring during pregnancy or after delivery? Please give an adequate discussion.

**Response:** Thanks for your good suggestion. We have added a paragraph for clarification in the third part of discussion.

**In the third part of the discussion:** “Low maternal vitamin D levels during pregnancy are associated with various adverse obstetric outcomes, such as maternal osteomalacia, gestational diabetes, preeclampsia, and cesarean section. Additionally, prenatal vitamin D deficiency is linked to fetal intrauterine growth restriction and a range of adverse fetal and neonatal health outcomes, including preterm birth, miscarriage, low birth weight, neonatal hypocalcemia, and an increased risk of childhood obesity.”

Special thanks to you for your good comments.

**Reviewer #3:** Summary

In this manuscript, Chen et al. performed a thorough meta-analysis and sensitivity analysis to examine the prevalence and potential factors of maternal vitamin D deficiency in mainland China. With a robust criterion for selecting high-quality literature, the manuscript provides a relatively accurate reflection of the statistics over the last decade (2013–2023). The regional variation in vitamin D deficiency highlights some facts about dietary dependence. It is also worth noting that, due to the limitations of the data, resources, and design of the study, some important features or observations were not clearly demonstrated. Overall, the manuscript is well-written. However, there are some flaws in the results that need to be addressed. Please refer to the comments outlined below. Please also proofread the manuscript and correct all noticeable grammatical errors.

Major Comments

• There is a lack of references being cited in the introduction section. As an illustration,
1. Line 48-52, the first paragraph: “Vitamin D is one of essential nutrients critical to metabolic processes in human body…. osteochondrosis.” Please add references.
2. Line 61, “The concentration of 25-(OH)D remained relatively stable in the whole process of pregnancy.”
3. Line 68-71, “Vitamin D regulation of the intrauterine… pregnancy complications.”
**Response:** Thank you very much for your reminder. We have added some references in the revised manuscript.

• For the Chi squared tests used for heterogeneity analysis, what would the results be if the authors choose a more common significance level alpha = 0.05?
**Response:** For the Chi squared tests used for heterogeneity analysis, if the level alpha = 0.05, the rate of false negatives will increase. For this study, the result would be more conservative.

• Based on the literature quality evaluation system, a score between 8 to 11 denotes high quality literature. According to the authors, the study only included the classification of high-quality literature. However, based on Table 1 AHRQ scores, 23 out of the 26 selected studies have a score that falls into the medium quality literature. I suggest the authors make a clearer clarification in section 2.3. For the meta-analysis, 88% of the studies were non-high-quality.
**Response:** Thank you very much for your care, and we apologize for our mistake. We have checked and revised the sentence in the revised manuscript.

**In the part of the Literature quality evaluation:** “The cross-sectional survey study used the quality evaluation tool recommended by the American Agency for Healthcare Research and Quality (AHRQ) [23], which includes 11 evaluation indicators. The options for each indicator are "yes", "no" and "unclear", among which " "Yes" is scored as 1 point, "No" and "Don't know" are both scored as 0 points when total score is 11 points. 8 to 11 denote high quality literature, 4 to 7 denote medium quality literature, and 0 to 3 denote low quality literature.”

• As the authors mentioned, one of the limitations of the analysis in the regional distribution comparison is the unbalanced number of studies conducted in different regions. They do differ quite a lot so the heterogeneity test might not be too convincing. Is there a way that the authors could subdivide the studies based on a better criterion? For instance, let the categorization be based on cities/provinces instead of national regions. This might improve the results in the heterogeneity test.
**Response:** Thanks very much for this good suggestion. We reviewed the included literature carefully, and we didn’t seen a better way.

• There is some inconsistency regarding the selection criteria. Based on figure 1, studies with population/sample size smaller than 1000 were excluded. However, there is one study in Table 1 that only has 953 population: Chun et al. 2021.
**Response:** Thank you very much for your care, and we apologize for our mistake. We have checked and revised the figure 1 in the revised manuscript.

• Although the authors concluded that there are no temporal effects on the vitamin D deficiency, the meta-analysis did not consider regional differences. I suggest the author perform the analyses within each regional subgroup.
**Response:** Thank you for your valuable feedback. We appreciate your point regarding regional differences. Due to data limitations, we were unable to conduct a detailed analysis of regional variations in our study. However, we will carefully consider your suggestion and plan to conduct subgroup analyses focusing on pregnant women from different regions in future research to explore the potential impact of regional differences on vitamin D deficiency. We are grateful for your insightful input and assure you that we will thoroughly consider your perspective in the revised manuscript.

• It seems less clear to me how to justify the selection criterion in the sensitivity analysis. The authors mentioned the small-sample study of Wu et al. [36] was excluded on Line 234. However, that study does not have the lowest sample size. It also has a higher incidence (%) than those lower ranked ones. Also reference 36 is not matching the one mentioned above:
[36] Xu YH, Qian YB, Zeng PP, et al. Analysis of the nutritional status of serum 25-hydroxyvitamin D in pregnant women in different seasons in Wuxi region. Hainan Medical Journal 2015;26:68-69.
Please make sure all cited references are in the correct order. Is it referring to [34]?
**Response:** Thank you for highlighting this issue. We apologize for the confusion regarding the reference to the study by Wu et al. [36]. Upon revisiting our selection criterion and conducting a thorough review, we have identified an error in referencing. The correct study with the smallest sample size is indeed Reference [21], which was excluded in the sensitivity analysis. We appreciate your attention to detail and will ensure that this correction is accurately reflected in the revised manuscript. Additionally, we will provide a clear and transparent justification for the selection criterion in the sensitivity analysis. Your feedback has been immensely valuable in improving the accuracy and clarity of our work.

• If there is only one study conducted in Northwest China, I find it less convincing or sound to conclude that “the highest incidence was found in Northwest China”. This is also one of the major limitations in the study. The same applies to “lowest incidence was observed in South Central China” with only one study included.

**Response:** Thanks very much for this good suggestion. We did not analyze the two regions separately in the revised manuscript.Furthermore, we have explicitly acknowledged in the limitations section that due to limited sample sizes in certain regions, we did not conduct further geographical analysis. We fully recognize the impact of this limitation on the study's conclusions and will give it due consideration in future research. We will also emphasize the applicability and limitations of the study findings in the discussion section.

**In the part of the 3.3 regional distribution:**Deleted“Specifically, the highest incidence was found in Northwest China, while the lowest incidence was observed in South Central China.”

**In the fourth part of the discussion:** “Our subgroup analysis revealed significant differences in the prevalence of vitamin D deficiency among pregnant women in different regions of China. This disparity can be attributed to the vast expanse of China and the substantial regional variations in dietary habits, lifestyles, and economic status. The identification of these regional disparities serves as a reminder for us to consider the influence of geographical factors when formulating policies for vitamin D supplementation in pregnant women. Targeted intervention measures should be implemented to address the vitamin D deficiency among pregnant women in different regions.”

**In the eighth part of the discussion:** “Secondly, in conducting regional subgroup analyses, the limited number of eligible literature resulted in some regions having only one included study and no further analysis in the geographical analysis, thereby increasing the uncertainty of the results and the level of uncertainty in the conclusions. ”

• I appreciate the authors mentioning the pivotal factors: “However, maternal vitamin D status can be influenced by multiple factors, like the mother's ethnicity, education level, body mass index (BMI) before pregnancy, sun exposure, pregnancy season, usage of vitamin D supplements as well as local customs regarding clothing habits.” I hope the authors could elaborate more on how these factors could possibly be associated with the regional differences as well as temporal effects on vitamin D deficiency. If necessary, some augmented analyses could also be included.
**Response:** Thanks very much for this good suggestion. We explain this in the first paragraph in the discussion.

**In the first part of the discussion:** “It's important to note that our current research did not collect specific data related to these factors, and therefore, we are unable to provide empirical evidence to support their contribution to the significant differences observed in our study*.*”

Minor comments
• Typo Line 130, “ ” Yes”.
• Line 66, improper use of comma. Please correct this run-on sentence. Consider rewriting or breaking it up.
• Grammatical error, Line 99-100. “The ultimate goal…”
• Line 103, missing period.
• Line 163, sentence is misleading. “The selection process refers to Fig 1.” Improper use of “refer to”. Please rephrase.
• Line 153, formatting issue after “random-effects model”.

**Response:** Thank you very much for your care, and we apologize for our mistake. We have checked and revised them in the revised manuscript.

Special thanks to reviewers for their good comments.

6. PLOS authors have the option to publish the peer review history of their article ([what does this mean?](https://journals.plos.org/plosone/s/editorial-and-peer-review-process#loc-peer-review-history)). If published, this will include your full peer review and any attached files.

If you choose “no”, your identity will remain anonymous but your review may still be made public.

**Do you want your identity to be public for this peer review?** For information about this choice, including consent withdrawal, please see our [Privacy Policy](https://www.plos.org/privacy-policy).

Reviewer #1: No

Reviewer #2: No

Reviewer #3: No

Special thanks to reviewers for their good comments.

We tried our best to improve the manuscript and made some changes in the manuscript. These changes will not influence the content and framework of the paper. And here we did not list the changes but marked in red in revised paper.

We appreciate for Editors/Reviewers’ warm work earnestly, and hope that the correction will meet with approval.

Once again, thank you very much for your comments and suggestions.

Yours sincerely

Zisheng Li
